# Giant Clams and Rising CO_2_: Light May Ameliorate Effects of Ocean Acidification on a Solar-Powered Animal

**DOI:** 10.1371/journal.pone.0128405

**Published:** 2015-06-17

**Authors:** Sue-Ann Watson

**Affiliations:** Australian Research Council Centre of Excellence for Coral Reef Studies & College of Marine and Environmental Sciences, James Cook University, Townsville, QLD, 4811, Australia; Universität Göttingen, GERMANY

## Abstract

Global climate change and ocean acidification pose a serious threat to marine life. Marine invertebrates are particularly susceptible to ocean acidification, especially highly calcareous taxa such as molluscs, echinoderms and corals. The largest of all bivalve molluscs, giant clams, are already threatened by a variety of local pressures, including overharvesting, and are in decline worldwide. Several giant clam species are listed as ‘Vulnerable’ on the IUCN Red List of Threatened Species and now climate change and ocean acidification pose an additional threat to their conservation. Unlike most other molluscs, giant clams are ‘solar-powered’ animals containing photosynthetic algal symbionts suggesting that light could influence the effects of ocean acidification on these vulnerable animals. In this study, juvenile fluted giant clams *Tridacna squamosa* were exposed to three levels of carbon dioxide (CO_2_) (control ~400, mid ~650 and high ~950 μatm) and light (photosynthetically active radiation 35, 65 and 304 μmol photons m^-2^ s^-1^). Elevated CO_2_ projected for the end of this century (~650 and ~950 μatm) reduced giant clam survival and growth at mid-light levels. However, effects of CO_2_ on survival were absent at high-light, with 100% survival across all CO_2_ levels. Effects of CO_2_ on growth of surviving clams were lessened, but not removed, at high-light levels. Shell growth and total animal mass gain were still reduced at high-CO_2_. This study demonstrates the potential for light to alleviate effects of ocean acidification on survival and growth in a threatened calcareous marine invertebrate. Managing water quality (e.g. turbidity and sedimentation) in coastal areas to maintain water clarity may help ameliorate some negative effects of ocean acidification on giant clams and potentially other solar-powered calcifiers, such as hard corals.

## Introduction

Carbon dioxide (CO_2_) emissions from fossil fuel combustion, industrial processes and large-scale land use changes are contributing to global change in the terrestrial and marine biospheres. Since the beginning of the Industrial Revolution, the oceans have absorbed approximately one third of all anthropogenic CO_2_ emissions released into the atmosphere [1, 2]. Consequently, the partial pressure of CO_2_ (*p*CO_2_) in the surface ocean is increasing in parallel with atmospheric CO_2_ [3]. In seawater, CO_2_ reacts to form carbonic acid and, as a result, surface oceans are now 0.1 pH units lower [4] and 30% more acidic [4, 5] than before the Industrial Revolution. This process is known as ocean acidification. Ocean chemistry is changing 100 times faster than any period in the last 650,000 years [6, 7] and projected changes in ocean pH are greater and far more rapid than any experienced in the last 24 million years [5] and possibly the last 300 million years [4]. Under current CO_2_ emission scenarios (RCP 8.5), atmospheric CO_2_ levels are projected to exceed 900 ppm by the end of this century [8] and seawater pH projected to decline a further 0.14–0.43 units [9]; the latter equivalent to ~150% increase in acidity [10].

Marine ecosystems are threatened by the increasing CO_2_ enrichment of the oceans [2, 3]. The effects of ocean acidification, including the decreasing saturation state of seawater with respect to calcium carbonate, pose particular threats to calcifying marine organisms because they affect the formation of calcium carbonate shells and skeletons [10–14]. In addition to calcification, ocean acidification can have a range of negative effects in calcifying invertebrates including reductions in survival and growth, as well as altered developmental and physiological processes (reviewed in [15–18]). In marine molluscs, ocean acidification has largely negative impacts on survival, growth, development and shell formation (e.g. [19–23]) and can also alter behaviour [24–26].

The world’s largest bivalve molluscs are giant clams [27], and the largest giant clam species, *Tridacna gigas*, can grow up to 1.3 m long, weigh up to 500 kg and produce the largest bivalve shell that has ever existed [28]. Icons of tropical coral reefs, giant clams are also an important economic and protein resource in the Indo-West Pacific [29]. However, giant clams are threatened by widespread overexploitation for meat and collection for the aquarium trade. Populations of most giant clam species are in decline [30], and some species are currently extinct in areas of their former range. Resultantly, all giant clam species are protected under the Convention of International Trade in Endangered Species of Wild Fauna and Flora (CITES) and are listed on the International Union for Conservation of Nature (IUCN) Red List of Threatened Species. Four tridacnid giant clams (*T*. *derasa*, *T*. *gigas*, *T*. *rosewateri* and *T*. *tevoroa*) are listed as ‘Vulnerable’ species meaning they face a ‘high risk of extinction in the wild’ [31]. Importantly, giant clams may have limited genetic connectivity, even in the global centre of marine biodiversity – the Coral Triangle – indicating that the genus *Tridacna* may be more endangered than currently recognised [29]. Now, in addition to local pressures such as overexploitation, giant clams are also threatened by global change including ocean acidification and ocean warming. Currently, little is known about the effects of global change on giant clams [32] and this knowledge gap limits the capacity to mitigate any impacts. Recent studies show ocean acidification and ocean warming may reduce survival in giant clams [33] and show seawater that is both high in nutrients and low in pH could have variable effects on growth [34]. However, any effects of ocean acidification in isolation on giant clam growth are unknown, and there are currently insufficient data to ascertain the likely impacts of ocean acidification on giant clam populations.

Unlike the majority of molluscs, giant clams form symbiotic associations with photobionts that capture light energy through photosynthesis. Oxygen and energy are produced allowing the host to survive in nutritionally poor habitats such as tropical oceanic waters [35]. Symbioses between heterotrophic animals and photoautotrophic algae have evolved in several taxa and metazoan examples include molluscs (giant clams, nudibranchs), cnidarians (corals, anemones, hydra), sponges, flatworms and ascidians [36] and the spotted salamander [37]. Since photosynthesis requires both light and CO_2_, light availability may influence the effects of ocean acidification on animals with photoautotrophic symbionts. Indeed, non-calcifying taxa, such as sea anemones could flourish at elevated CO_2_ [38]. However, for calcifying taxa such as giant clams and corals, the influence of light with rising CO_2_ may be particularly important. Among calcareous animals the effects of light on ocean acidification have been previously investigated in only three studies that examined four species of hard coral. Reduced seawater pH (by HCl addition) decreased calcification at all light levels (81–698 μmol photons m^-2^ s^-1^) in *Porites compressa* nubbins [39]. In *Acropora horrida* and *Porites cylindrica* nubbins held at 75–600 μmol photons m^-2^ s^-1^, daytime calcification losses at elevated CO_2_ were greatest in low light [40]. In *Pocillopora damicornis* recruits held at 14–226 μmol photons m^-2^ s^-1^, elevated CO_2_ reduced calcification at intermediate light levels, although results varied, and neither elevated CO_2_ nor light had a clear trend on recruit survival [41]. While results for corals may vary, nothing is known of the potential influence of light on ocean acidification effects in other calcifiers with photoautotrophic symbionts, such as giant clams.

Understanding the potential interaction of global change and light availability in marine animals with photoautotrophic symbionts is particularly important in coastal areas where local human disturbances can also affect marine ecosystems. Light levels can be reduced by turbidity and sedimentation, potentially exacerbating effects of global change on coastal marine organisms. Consideration of these factors may be particularly important in conservation efforts and the management of threatened marine species with continuing human development along coastlines. To determine if near-future CO_2_ levels affect giant clam survival and growth, and any potential influence of light availability, I conducted a series of experiments on the fluted giant clam *Tridacna squamosa* at 3 CO_2_ levels and 3 light levels. It was predicted that CO_2_ and light availability may alter mortality and growth rates in giant clams, and specifically that increasing CO_2_ may reduce survival and growth whereas increasing light may ameliorate negative effects on survival and growth.

## Materials and Methods

### Study species

The fluted, or scaly, giant clam, *Tridacna squamosa* Lamarck, 1819, listed as ‘Lower Risk/conservation dependent’ on the IUCN Red List of Threatened Species [31], is native to shallow coral reefs of the South Pacific and Indian Oceans, but possibly extinct in Japan and the Northern Mariana Islands [31]. A new species range extension into French Polynesia has been observed recently [42], although this may be a relic [43]. The fluted giant clam has leaf-like shell protrusions called scutes and is one of the most ornate giant clam species. Like ornamentation in other molluscs, scutes may be an antipredator adaptation as they increase overall shell size reducing the number of potential crushing and grasping predators [44]. In this study, *T*. *squamosa* were spawned from wild caught broodstock at the Darwin Aquaculture Centre, Australia. Broodstock were collected under Special Permit No. 2007-2008/S17/2441 issued under the Northern Territory *Fisheries Act 1998*.

### Experimental systems and CO_2_ manipulation

Juvenile *T*. *squamosa* were transported to the James Cook University aquarium facility where they were kept in natural seawater sourced from the Australian Institute of Marine Science seawater intake facility at nearby Cape Cleveland. This natural seawater was filtered to 1 μm and UV sterilised before introduction into the aquarium systems. In the aquarium facility, giant clams gained energy both from photoautotrophic algal symbionts as well as from some heterotrophic filter feeding on microorganisms present in the aquarium systems, and supplemental feeding was not undertaken. Three >8,000 l recirculating seawater systems were maintained at three different partial pressures of carbon dioxide (*p*CO_2_): 1) current-day control-, 2) mid- and 3) high-CO_2_. Mean (±s.e.) CO_2_ levels for the three experiments combined were: 1) present-day control *p*CO_2_ 400 ± 8 (range 387–410) μatm; 2) mid *p*CO_2_ 661 ± 15 (range 616–704) μatm; and 3) high *p*CO_2_ 937 ± 23 (range 890–992) μatm. CO_2_ levels for each experiment are reported in Table 1. These elevated CO_2_ treatments are consistent with projections of CO_2_ in the ocean over the next 50–100 years [8]. Elevated CO_2_ treatments were achieved by dosing 100% CO_2_ into a 3,000 l temperature-controlled sump on each system to a set pH using a pH control system (AT-Control, Aqua Medic, Germany) following standard techniques [45]. Seawater was maintained at a mean temperature of 28.3 ± 0.0°C (±s.e.) by a heater-chiller on each system before delivery to individual aquaria at a flow rate of 400 ml.min^-1^. Temperature (C22, Comark, Norwich, U.K.) and pH_NBS_ (HQ40d, Hach, Colorado, U.S.) were recorded daily in the treatment tanks and seawater CO_2_ confirmed with a portable CO_2_ equilibrator and non-dispersive infra-red (NDIR) sensor (GMP343, Vaisala, Helsinki, Finland). Salinity and total alkalinity were measured weekly. Total alkalinity was analysed by Gran titration from water samples of replicate tanks in each system to within 1% of certified reference material (Prof. A.G. Dickson, Scripps Institution of Oceanography). Seawater *p*CO_2_ was calculated in the program CO2SYS [46] using the constants of Mehrbach *et al*. 1973 refit by Dickson & Millero 1987, and Dickson for K(HSO_4_^-^). Seawater carbonate chemistry parameters are provided in Table 1.

**Table 1:** Seawater carbonate chemistry data (mean ± s.e.) and experimental conditions.

CO_2_ condition	PAR (μmol photons m^-2^ s^-1^)	Light type and photoperiod	Temperature (°C)	Salinity	pH_NBS_	Total alkalinity (μmol.kg^-1^ SW)	*p*CO_2_ (μatm)	Ω_Ca_	Ω_Ar_	Tank volume (l)	No. of individuals per tank	No. of replicate tanks	Total no. of individuals
Control-CO_2_	Low ~35: 34.0 (±4.4)	Tri-phosphor T8 13L:11D	28.4 (±0.1)	34.2 (±0.2)	8.23 (±0.00)	2578.6 (±9.4)	387.4 (±5.7)	6.97 (±0.08)	4.64 (±0.05)	2	2	8	16
Mid-CO_2_	Low ~35: 32.9 (±3.7)	Tri-phosphor T8 13L:11D	28.5 (±0.1)	34.0 (±0.2)	8.02 (±0.01)	2317.6 (±10.9)	664.7 (±15.0)	4.20 (±0.09)	2.79 (±0.06)	2	2	8	16
High-CO_2_	Low ~35: 38.5 (±4.7)	Tri-phosphor T8 13L:11D	28.5 (±0.0)	34.7 (±0.1)	7.93 (±0.01)	2435.5 (±10.2)	889.8 (±20.7)	3.76 (±0.10)	2.50 (±0.06)	2	2	8	16
Control-CO_2_	Mid ~65: 64.6 (±6.2)	Tri-phosphor T8 13L:11D	28.4 (±0.0)	32.5 (±0.2)	8.15 (±0.01)	2045.3 (±13.4)	409.8 (± 9.4)	4.53 (±0.11)	3.00 (±0.07)	40	2	8	16
Mid-CO_2_	Mid ~65: 71.1 (±2.7)	Tri-phosphor T8 13L:11D	28.5 (±0.0)	33.0 (±0.1)	8.01 (±0.01)	2103.4 (±7.8)	615.6 (± 11.8)	3.61 (±0.06)	2.39 (±0.04)	40	2	8	16
High-CO_2_	Mid ~65: 59.5 (±7.8)	Tri-phosphor T8 13L:11D	28.5 (±0.0)	33.1 (±0.1)	7.84 (±0.01)	2170.6 (±7.1)	992.0 (± 23.7)	2.68 (±0.07)	1.77 (±0.05)	40	2	8	16
Control-CO_2_	High ~304: 310.3 (±11.5)	Filtered light 11-12L:12-13D	28.0 (±0.1)	34.2 (±0.2)	8.24 (±0.01)	2645.0 (±3.0)	401.0 (±8.0)	7.05 (±0.08)	4.68 (±0.05)	2	1	20	20
Mid-CO_2_	High ~304: 290.0 (±12.2)	Filtered light 11-12L:12-13D	27.9 (±0.1)	33.6 (±0.3)	8.01 (±0.01)	2413.4 (±2.7)	703.7 (±17.5)	4.21 (±0.08)	2.79 (±0.06)	2	1	20	20
High-CO_2_	High ~304: 312.2 (±13.3)	Filtered light 11-12L:12-13D	27.9 (±0.1)	34.4 (±0.1)	7.92 (±0.01)	2524.1 (±3.9)	928.3 (±23.3)	3.76 (±0.08)	2.50 (±0.06)	2	1	20	20

For seawater carbonate chemistry data, temperature, salinity, pH_NBS_ and total alkalinity (TA) were measured directly. *p*CO_2_, Ω_Ca_, Ω_Ar_ were estimated from these parameters using CO2SYS. Opportunistic use of aquarium space on CO_2_ systems determined tank availability at each light level.

CO_2_ experiments were conducted at three different light levels on these systems. Light treatments were measured by photosynthetically active radiation (PAR, wavelength range 400–700 nm) and achieved by tri-phosphor T8 linear fluorescent lights and filtered natural light under a polycarbonate sheet by making opportunistic use of available aquarium space and tanks under different PAR regimes on the same large (>8,000 l) CO_2_-treated seawater systems. PAR was measured with a LI-COR LI-250A light meter and LI-COR LI-192SA Underwater Quantum Sensor meter. Mean PAR conditions were 1) low-light (PAR 35.1 ± 2.4 μmol photons m^-2^ s^-1^ (±s.e.)), 2) mid-light (PAR 65.1 ± 3.5 μmol photons m^-2^ s^-1^), and 3) high-light (PAR 304.2 ± 7.1 μmol photons m^-2^ s^-1^). The light levels were equivalent to ~5% (5.8%), ~10% (10.7%) and 50% ambient PAR, respectively and represented a range of light levels recorded on coral reefs [47–49]. PAR levels were significantly different among the three experiments (ANOVA on ranks H_2_ = 70.970, p<0.001), but PAR levels did not differ among CO_2_ conditions within each experiment (low-light F_2,33_ = 0.474, p = 0.627; mid-light F_2,24_ = 0.943; p = 0.403, high-light F_2,24_ = 0.994, p = 0.385). Since the experiments at different light levels were achieved on an opportunistic basis, full details of experiments are provided in Table 1. In all tanks, juveniles had ample space with more than two body lengths between individuals and never shaded each other. Pilot experiments at control-CO_2_ showed stocking density and tank size used did not affect survival. The two highest stocking densities of 1 (n = 20 tanks) or 2 (n = 19 tanks) clams per 2 l tank both resulted in 100% survival at high-light levels after 8 weeks (Mann-Whitney U = 590.000, n = 20,38, p = 2.000), and tank sizes of 2 l (n = 20 tanks) and 40 l (n = 8 tanks) also both resulted in 100% survival after 8 weeks (U = 270.000, n = 15,20, p = 1.000).

### Data collection

**Fig 1:**
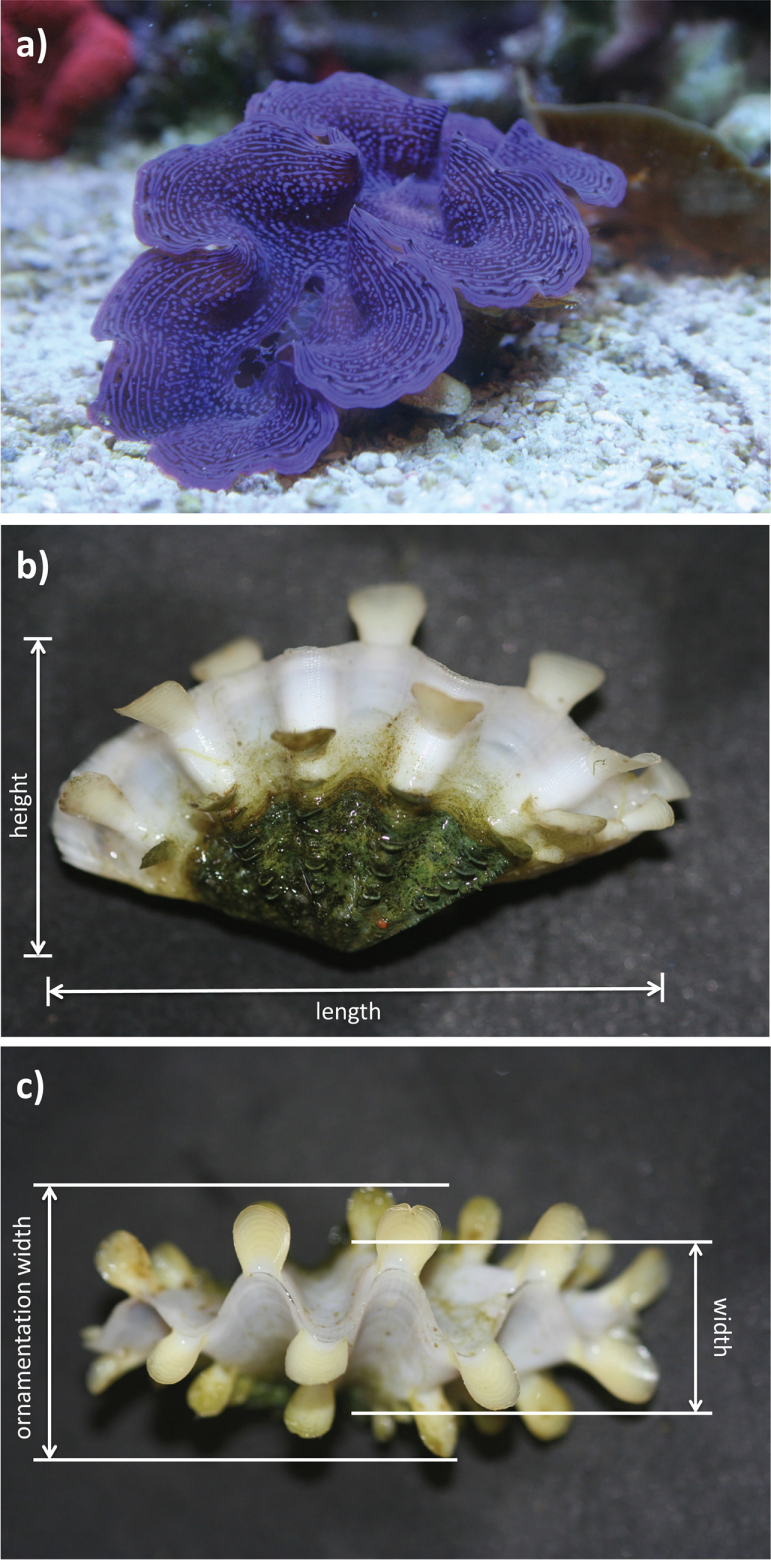
Giant clam shell measurements. Fluted giant clam *Tridacna squamosa* juvenile a) with mantle out, and b) and c) showing shell dimensions measured: length (anterior-posterior measurement), height (dorso-ventral measurement), width including ornamentation and width excluding ornamentation.

Individual giant clam shell dimensions (to 0.01 mm) and whole or total animal (soft tissues + shell) wet mass (to 0.0001 g) were measured before and after the experiment. Shell dimensions recorded included 1) shell length (anterior-posterior measurement) (see [50] for bivalve shell terminology), 2) shell height (dorsal-ventral measurement), 3) shell width (excluding ornamentation) and 4) shell ornamentation width (the total width of shell including projecting scutes at widest point) (Fig 1). Giant clam individuals were assigned randomly to the 3 CO_2_ levels across 3 light levels. At the start of the experiment, mean shell length was 22.3 ± 0.4 mm (±s.e.) and total animal wet mass was 1.144 ± 0.051 g. Giant clams were held in experimental treatment conditions for 8 weeks (56 d) after which shell dimensions and total animal wet mass were re-measured for all surviving clams. Any dead clams were recorded and removed immediately from the experiment.

### Data analysis

Data were analysed both across all CO_2_ and light levels (3 CO_2_ x 3 light experimental design) and for CO_2_ at each light level (3 CO_2_ x 1 light experimental design) using TIBCO Spotfire S+ 8.2 and SigmaPlot 11.0. A logistic regression was performed to compare the effects of CO_2_ and light (3 CO_2_ x 3 light levels) among experiments on the final proportion of survivors after 8 weeks in treatment conditions. Additionally, at each light-level, survival trajectories (up to 56 d) of giant clams among the 3 CO_2_ levels (control, mid and high) were compared using Kaplan-Meier Log-Rank Survival Analysis.

Growth gains were calculated from initial and final size measurements as a percentage of initial size to allow for small differences in the initial size of individual giant clams. A linear mixed effects (LME) model on total animal mass gain with CO_2_ and light as fixed effects, tank as a random effect and allowing for heterogeneous variance among light levels was conducted to determine any potential interaction of CO_2_ and light on total animal growth. Measures of shell growth (shell length, height, width and ornamentation width gains) were all highly correlated with each other (correlation coefficients between pairs of measures varied from 0.92 to 0.96). Consequently a principal component analysis was used to generate an overall measure of shell growth as the first principal component, and this aggregated measure of shell growth was analysed in the same way as total animal mass gain. Analysis of variance (ANOVA) or Kruskal-Wallis ANOVA on ranks followed by Holm-Sidak or Dunn’s pairwise multiple comparison procedures, respectively, were conducted at each light level to indicate where growth variables in elevated-CO_2_ treatments differed from control-CO_2_ conditions. Akaike information criterion (AIC), likelihood ratio tests and residual analysis were used to examine model fit and assumptions of analyses.

## Results

### Survival

The proportion of giant clam juveniles that survived was affected by CO_2_ (p = 0.038) and light (p<0.001), but no interaction was detected between CO_2_ and light on survival (S1 Table). After 8 weeks at low-light levels, some mortality occurred at all 3 CO_2_ levels. With increasing CO_2_ in the low-light treatment there was a decreasing trend in survival from 81.3% in control- to 75.0% in mid- and 68.8% in high-CO_2_ conditions. At mid-light, 100% of giant clams survived in control-CO_2_, however, survival decreased to 75.0% at mid- and 53.3% at high-CO_2_. Conversely, at high-light conditions, survival was 100% at all CO_2_ levels.

**Fig 2:**
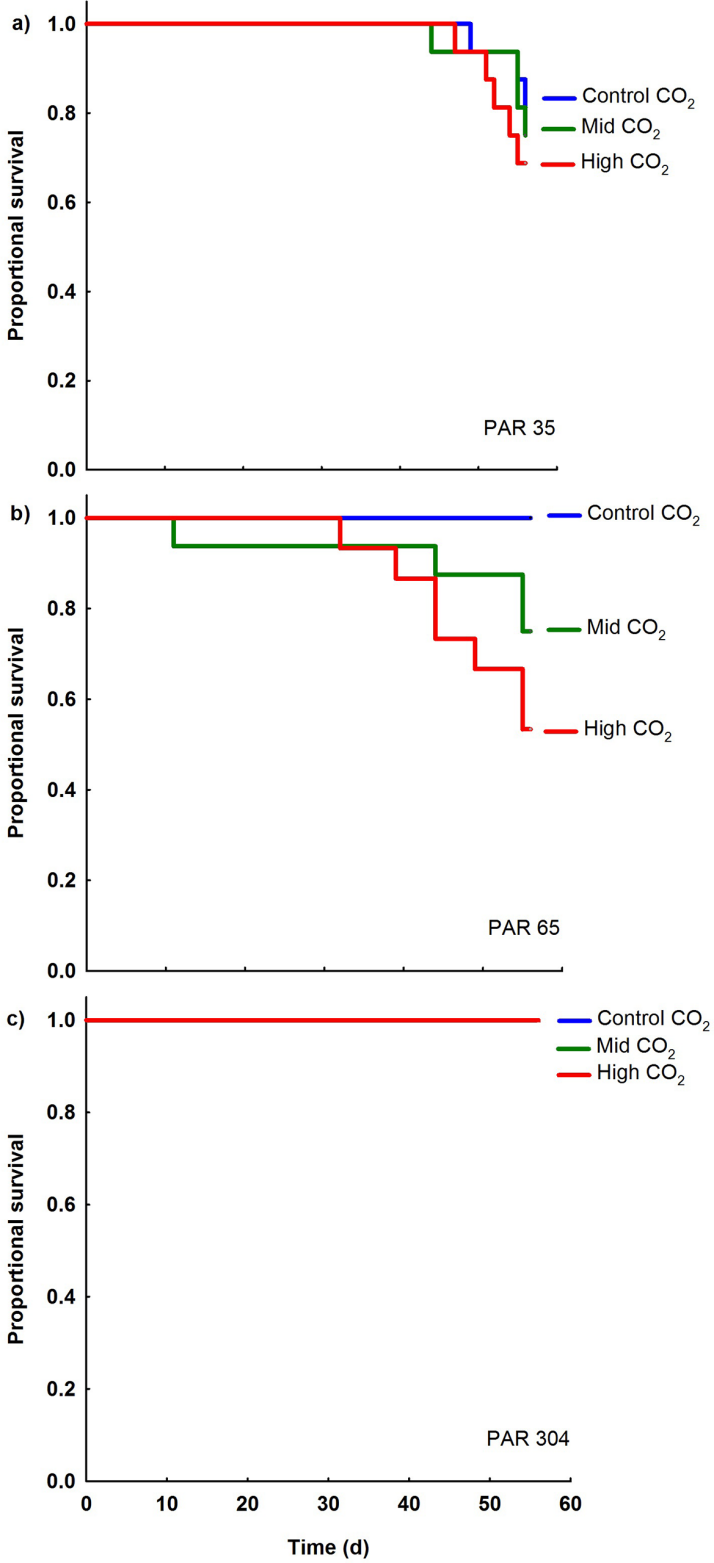
Influence of elevated CO_2_ on giant clam survival at each light level. Effects of CO_**2**_ on juvenile fluted giant clam survival shown by Kaplan-Meier survival trajectories at a) low-light (PAR 35 μmol photons m^-2^ s^-1^), b) mid-light (PAR 65 μmol photons m^-2^ s^-1^) and c) high-light (PAR 304 μmol photons m^-2^ s^-1^). At high-light, survival was 100% so survival trajectories are the same for all CO_**2**_ levels.

There was no significant difference in survival trajectories among the three CO_2_ levels in the low-light experiment (χ^2^ = 0.854, df = 2, p = 0.653, Fig 2A, S2 Table). In the mid-light experiment, elevated CO_2_ reduced survival (χ^2^ = 7.106, df = 2, p = 0.029, Fig 2B, S2 Table), with mortality increased at mid- (p = 0.042) and high-CO_2_ (p = 0.003) (S3 Table). In the high-light experiment, mortality was 0% (Fig 2C) so survival analysis was not conducted.

### Growth

**Fig 3:**
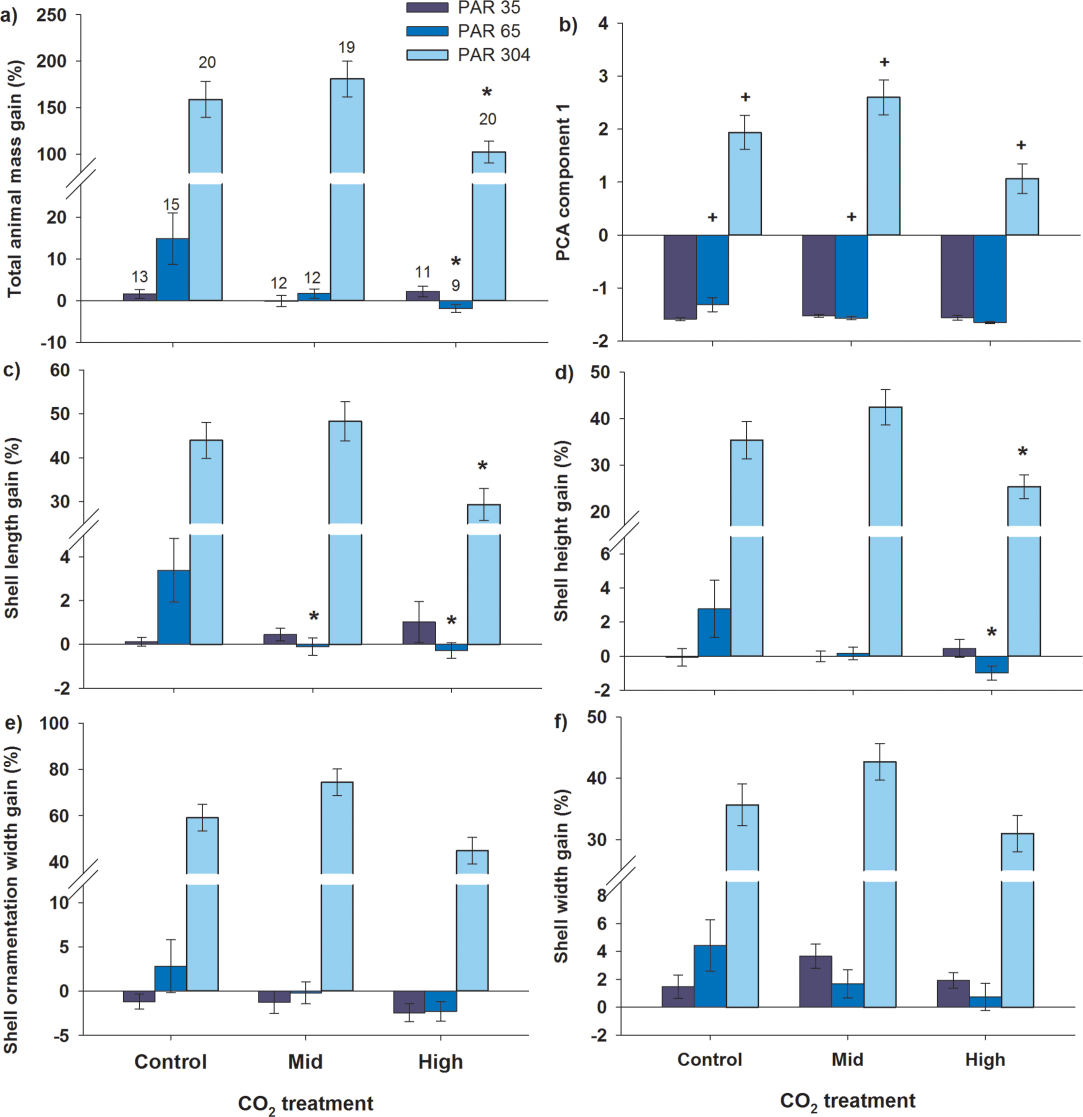
Influence of elevated CO_2_ and light on giant clam growth. Effects of CO_**2**_ and PAR on juvenile fluted giant clam growth in a) total animal mass, b) principle component analysis (PCA) component 1 (i.e. all shell linear dimensions), and individually, c) shell length, d) shell height, e) shell ornamentation width, and f) shell width gains. Numbers of replicates are the same for each graph and are shown above the bars in a). *denotes a significant difference from the control at each light level (for a, c-f). ^+^denotes a significant difference from the PAR 35 control-CO_**2**_ level in PCA component 1 (for b). Error bars represent ±1 s.e.

There were significant interactions of CO_2_ and light on total animal mass gained (F_4,94_ = 4.632, p = 0.002, Fig 3A, S4 Table) and principle component (PC) 1 (F_4,94_ = 4.834, p = 0.001, Fig 3B, S5 Table), which summarised shell growth (shell length, height, width and ornamentation width gained). PC1 accounted for 96% of the total variance in shell growth morphology measurements (S1 Fig). Neither CO_2_ nor light had any effect on PC2, which accounted for a further 2% of shell morphology (S5 Table).

Light had a large effect on total animal and shell growth, with growth at the mid-light level approximately an order of magnitude or greater than growth at low-light, and growth at the high-light level an order of magnitude greater again than growth at mid-light across all CO_2_ levels (Fig 3). At low-light, all measures of growth were minimal and there were no differences among CO_2_ treatments. In contrast, high-CO_2_ reduced mass gain at the mid-light (112.8% decrease, p<0.05) and high-light (35.7% decrease, p<0.05) levels compared with control-CO_2_ (Fig 3A, S6 Table). Negative growth in mass was exhibited at high-CO_2_ at the mid-light level, since decreases >100% equate to negative growth.

Shell growth at low-light was not affected by CO_2_ (Fig 3C–3F). However, elevated CO_2_ reduced growth at mid- and high-light levels. At mid-CO_2_, shell length growth was reduced at the mid-light level (103.3% decrease, p<0.05) compared with control-CO_2_ (Fig 3C). At high-CO_2_, shell growth was reduced at the mid-light (shell length 108.3% decrease, shell height 135.6% decrease) and high-light (shell length 33.3% decrease, shell height 28.3% decrease) levels compared with control-CO_2_ at each respective light level (all p<0.05) (Fig 3C and 3D, S6 Table). Negative growth was exhibited at mid-light conditions at one or more elevated CO_2_ levels for shell length, height and ornamentation width, and at low-light for shell ornamentation width across all CO_2_ levels. No significant differences were detected in shell width or ornamentation width gains among CO_2_ levels, although there was a trend for reduced gains in shell width and ornamentation width with increasing CO_2_ at the mid-light level (Fig 3E and 3F).

## Discussion

Giant clams are currently threatened by a variety of local pressures and are listed on the IUCN Red List of Threatened Species. This study showed that giant clams are additionally threatened by ocean acidification, since elevated CO_2_ reduced survival and growth in juveniles of the fluted giant clam *Tridacna squamosa*. However, the magnitude of ocean acidification effects varied according to light level and some negative effects were ameliorated at higher light levels. In the mid-light (PAR 65 μmol photons m^-2^ s^-1^) experiment, giant clam survival was 100% in control CO_2_ conditions (~400 μatm), but was reduced with increasing CO_2_. Among surviving clams, shell growth was reduced at both mid- (~650 μatm) and high-CO_2_ (~950 μatm), and total animal mass gain was reduced at high-CO_2_. Negative growth in both total animal mass and shell size was observed at high-CO_2_. This mid-light level is likely to be close to marginal for *T*. *squamosa* survival and growth since the addition of elevated CO_2_ resulted in mortality and reduced growth. Negative growth at elevated CO_2_ in live weight and shell length of the venus clam has also been observed, although at quite high *p*CO_2_ (~2650 μatm) and low pH (7.38) seawater that was undersaturated with respect to aragonite [51]. In such undersaturated conditions, shell dissolution is likely to contribute to decreases in shell length. In the current study, although seawater was not undersaturated with respect to calcite or aragonite, shells appeared more fragile at high-CO_2_ and delicate shell edges and scutes could have eroded or broken as juvenile clams moved using their foot, resulting in negative shell growth. Reductions in total animal live mass could have resulted from both reductions in shell and soft tissue mass, the latter potentially through increased energy budget demands at high-CO_2_ conditions.

In the low-light study (PAR 35 μmol photons m^-2^ s^-1^), survival and growth were reduced at all CO_2_ levels, although there was still a trend of higher mortality with increasing CO_2_. This low-light level was very marginal for *T*. *squamosa* with reduced survival and almost no growth in surviving clams. Notably, in the experiment conducted at high-light (PAR 304 μmol photons m^-2^ s^-1^), giant clam survival was 100% at all 3 CO_2_ levels, suggesting that increased light may remove negative effects of ocean acidification on survival. At high-light, however, shell growth and total animal mass gain were both still reduced at high-CO_2_.

**Fig 4:**
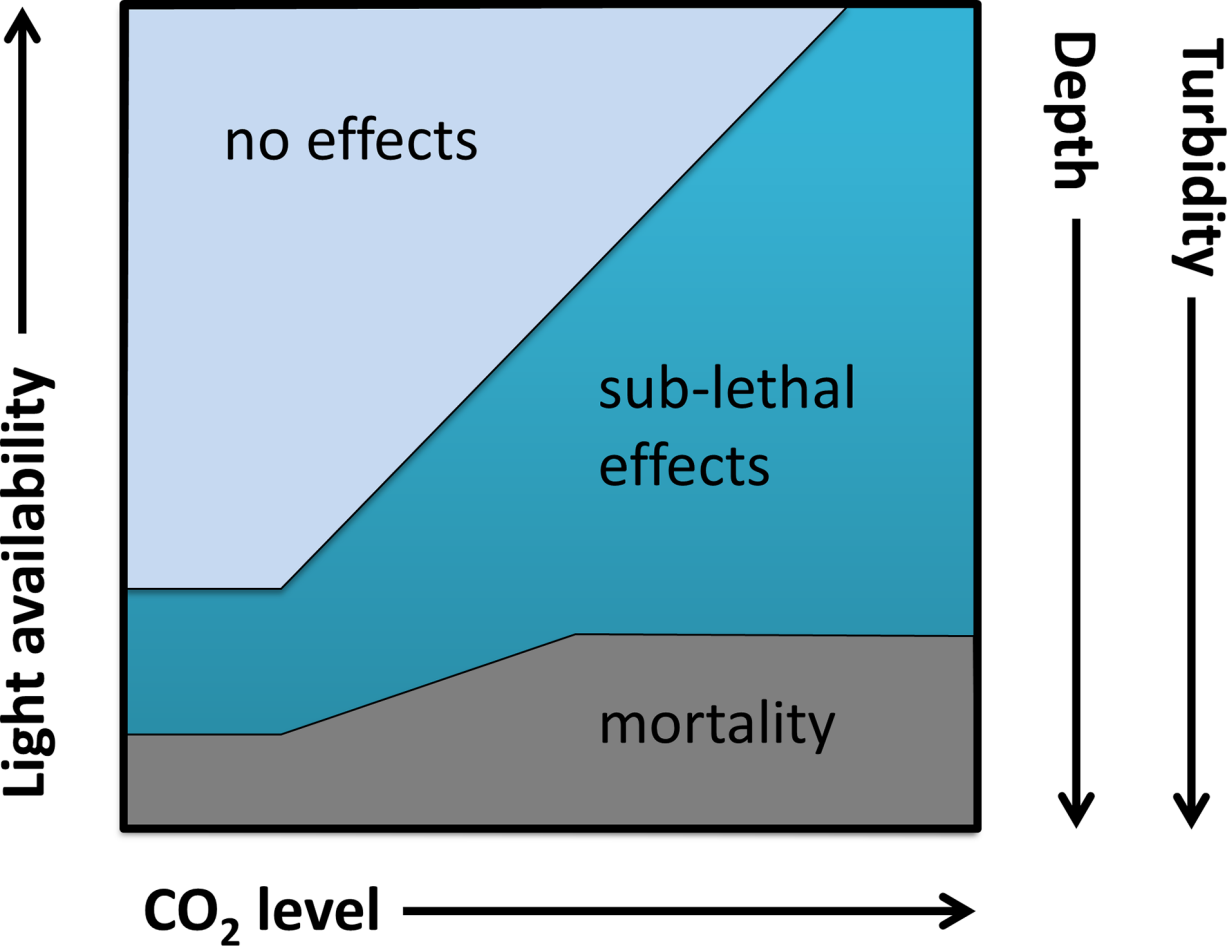
Conceptual diagram showing the relationship between CO_2_ and light availability. Seawater CO_**2**_ level (ocean acidification) and light availability influence the likelihood of sub-lethal and lethal effects on juvenile giant clams. This diagram is based on experimental data and is therefore for the range of light levels investigated this study only (PAR 35–304 μmol photons m^-2^ s^-1^).

Increasing light conditions appeared to ameliorate both lethal and sub-lethal effects of ocean acidification. Although aquarium facilities at different light levels were used opportunistically (experimental details in Table 1), the correlations between light levels and ocean acidification effects are compelling. The results of these experiments suggest that light may lessen some of the negative effects of elevated CO_2_ on giant clam survival and growth. For example, at high-light, ocean acidification no longer compromised survival and the effect on growth was absent, except at the highest CO_2_ – a level projected for the year 2100 (RCP 8.5) [8]. The combination of light and ocean acidification appeared to produce an antagonistic (reduced stress) response [52], suggesting that between PAR 35–304 μmol photons m^-2^ s^-1^, enhanced light availability may ameliorate ocean acidification effects. A conceptual model was constructed from the results of this study (Fig 4). This schematic diagram shows both the lethal and sub-lethal effects of rising CO_2_ on giant clams, and how the negative effects of rising CO_2_ could be influenced by increasing light availability. Additionally, other environmental factors such as depth and turbidity that act to reduce light availability are likely to influence responses to ocean acidification.

Giant clams are heterotrophs with photoautotrophic dinoflagellate symbionts within their tissues. As predicted, giant clam life-history traits improved with light in this study. Previous studies at ambient control-CO_2_ conditions show that light influences giant clam growth. Between ~12–50% ambient PAR (180–800 μmol photons m^-2^ s^-1^), Guest *et al*. [53] found the greatest *T*. *squamosa* shell length growth occurred at 800 μmol photons m^-2^ s^-1^. Another study found, across 0–80% shade that shell length and total animal wet mass of juvenile *T*. *squamosa* was greater in unshaded clams than at 55 and 80% and 10, 55 and 80% shade, respectively. However, there were no differences in juvenile *T*. *squamosa* survival under any of the light levels (0–80% shade) [54]. The 80% shade regime used in Adams *et al*. [54] likely had higher PAR conditions than the low-light level used in the current study, where survival was reduced at control-CO_2_.

Previous studies on hard corals, each using a single light intensity, show ocean acidification effects vary. This variation in ocean acidification responses among corals may be influenced by the variation in light intensities used that ranged from <10 to 700 μmol photons m^-2^ s^-1^ (reviewed in [41]). Although light intensities are not always reported for coral studies, they can often be low [41], and could result in increased negative findings from ocean acidification studies. In the three previous studies where ocean acidification effects across a range of light levels were tested on corals, there also appears to be different responses among taxa or life-stage. Reduced pH (by HCl addition) affected calcification in *Porites compressa* nubbins across all light levels suggesting elevated CO_2_ could affect this coral at all depths [39], but results for nubbins and recruits of other species appear to vary (see [40, 41]). The results of Suggett *et al*. [40] who found, between 75–600 μmol photons m^-2^ s^-1^, low light conditions resulted in the greatest ocean acidification related coral calcification losses in the light, are similar to findings in the current study of reduced tolerance to ocean acidification in giant clams at lower light levels.

Like corals, adult giant clams are sessile on the reef and their depth ranges are dictated by habitat suitability and light availability. Animals with photoautotrophic symbionts are limited in their upper depth distribution by excessive light (PAR levels beyond those used in this study), and the resultant increase in temperature which can limit photosynthesis and cause bleaching. On coral reefs, PAR reduces naturally with depth as light is attenuated in the water column and in clear tropical waters, light intensity is reduced by approximately 70–80% from the surface to 10 m depth [55]. In the marine environment, light levels are often very variable. Light levels on the Great Barrier Reef (GBR) can be around 520 and 250 μmol photons m^-2^ s^-1^ at 7 and 14 m depth, respectively, near One Tree Island (M. Hoogenboom, unpublished data, cited in [47]). These light levels are in the range of the high-light level used in the current study. However, on the central inshore GBR around the Palm Island Group, light at 3.5 m depth can average 70–180 μmol photons m^-2^ s^-1^ [48]. Light levels on these inshore reefs are just over the mid-light level used in the current study; light levels at slightly deeper depths are likely to approximate the mid-light levels used here.

Clams compete for space and light with other reef organisms including coral and algae. Because of the potentially enhanced requirements for light with ocean acidification (Fig 4), reduced survival at a given depth could mean giant clam depth ranges may shoal upwards. The depth range of *T*. *squamosa* is currently 0–25.5 m [56] with only a living relic *T*. *mbalavuana* (= *T*. *tevoroa*) found at greater depths [43]. With one of the deepest depth ranges of all giant clam species, *T*. *squamosa* may be able to tolerate some of the lowest light conditions. This could indicate that the lethal and sub-lethal effects of ocean acidification (Fig 4) may be worse for a given light level in other giant clam species. If upper depth distributions are already at their limit, ocean acidification may narrow the depth distribution of giant clams, consequently reducing the range of suitable habitat on coral reefs.

In addition to depth, several other factors affect light levels in the marine environment including cloud cover, turbidity and sedimentation. Natural events as well as regional or local anthropogenic impacts including land-use changes for urbanisation, coastal development, and agriculture in water catchment areas can result in increased turbidity and sedimentation in coastal marine environments. Turbidity increases light attenuation [57] and can lead to a dramatic reduction of light with depth. Singapore, for example, has a highly altered marine environment with high turbidity and sedimentation [58]. On coral reefs around Singapore, PAR at ~2 m depth can be >20% of surface PAR but reduces dramatically to <1% at ~9 m depth [49]. These reduced light levels may approximate the lower light levels in the current study. Turbidity and sedimentation have negative effects on coral reefs including increased prevalence of coral disease [58, 59]. Giant clam growth is negatively correlated with turbidity [60, 61] and *T*. *squamosa* exhibits increased activity in response to higher sediment loads with likely increased demands on the energy budget [62]. High turbidity and lack of suitable habitat create poor environmental conditions for giant clam reproduction and recruitment [63] and high sediment levels may hinder settlement and survival of larvae [53]. Such recruitment constraints on a sparse and scattered population are likely to inhibit recovery of natural giant clam populations [64]. Local management to ensure good water quality may help ensure the future persistence of threatened giant clams, and other species with photoautotrophic symbionts, including corals. Shifting the conceptual relationship in Fig 4 to maintain light levels through water quality management, may be important for the conservation of giant clam populations as CO_2_ rises this century.

An important additional consideration in the conservation of giant clams is their longevity compared to the rate of global change. Although calcareous marine invertebrates can adapt total shell size and morphology over evolutionary time in environments where the saturation state of calcium carbonate is reduced [65], the current rate of change in ocean chemistry is 100 times faster than at any time during the last 650,000 years [6, 7] with projected changes greater and far more rapid than possibly the last 300 million years [4]. These rates of change could outpace the rate of biological adaptation. Furthermore, giant clam life-history traits such as longevity and long times to maturity, infer that their scope for acclimation and adaptation to global change might be reduced relative to other species. Since giant clams may live for several decades [28, 30], present-day recruits could live long enough to experience ocean conditions late this century; however, any early-life developmental acclimation exhibited would be to present-day environmental conditions only.

This study focussed on whole-animal biology and investigated shell growth and mass gain as net products of calcification and photosynthesis. Further work could investigate the processes of calcification, metabolism, photosynthesis, symbiodinium characteristics, and other responses that may be altered by CO_2_ and light, and any potential interaction with elevated temperature. In the blue mussel, enhanced food supply and therefore energy availability increases tolerance to elevated CO_2_ [66, 67]. In giant clams, both increased light and food supply can enhance energy availability, and potentially increase tolerance to ocean acidification. Higher light levels could ameliorate CO_2_ effects through enhanced energy availability from photosynthetic symbionts providing a survival and growth advantage, and higher light levels could boost light-enhanced calcification. At suboptimal light levels, reduced symbiont photosynthesis could result in a net reduction in available energy and result in decreased growth and survival given constant food conditions. However, if food availability is enhanced, some negative effects of increased CO_2_ or reduced light could be lessened through enhanced heterotrophic nutrition.

Giant clams are currently threatened by a variety of local pressures and several species are already listed as ‘Vulnerable’ on the IUCN Red List of Threatened Species. Now giant clams are also threatened by global change including ocean acidification, which can reduce survival and growth. However, the potential for light to ameliorate negative effects of ocean acidification on giant clams may allow management intervention. As global change progresses during this century, local management could become increasingly important for giant clams and potentially other solar-powered marine calcifiers, including corals. Given that scope for adaptation in giant clams is likely to be reduced relative to other coral reef species, a focus on management of local as well as global press-type stressors [68] is likely to be important in ensuring the conservation of threatened giant clams into the future.

## Supporting Information

S1 Fig Principle component scree plot for shell growth morphology variables. (PDF)

S1 Table Logistic regression Chi-squared results on the influence of CO_2_ and light (PAR) on the proportion of survivors after 8 weeks. *denotes a significant result.
(PDF)

S2 Table Survival analysis results from Kaplan-Meier Log-Rank Survival Analyses on CO_2_ levels at each light (PAR) condition. *denotes a significant result. Tests were not conducted at PAR 304 as there were no deaths.
(PDF)

S3 Table Pairwise multiple comparisons from Kaplan-Meier Log-Rank Survival Analysis for CO_2_ levels at mid-light PAR 65. *denotes a significant result.
(PDF)

S4 Table Linear mixed effects (LME) model results. LME results on total animal (soft tissues + shell) wet mass gain.
(PDF)

S5 Table Principle component analysis (PCA) results. PCA results for shell growth morphology (shell linear dimensions: shell length, height, width and ornamentation width gains) and linear mixed effects model (LME) results on principle component (PC) 1. LME on PC2 was non-significant.
(PDF)

S6 Table Growth gain ANOVA results and pairwise multiple comparisons for experiments at each light level. *denotes a significant result.
(PDF)
