# Supplementary material for: Giant Clams and Rising CO2: Light May Ameliorate Effects of Ocean Acidification on a Solar-Powered Animal
Source: PLoS One. 2015 Jun 17;10(6):e0128405. doi: 10.1371/journal.pone.0128405 (PMC4470504; doi:10.1371/journal.pone.0128405)
Supplement: S1 Table — *denotes a significant result. (PDF) [file pone.0128405.s002.pdf]

Giant clams and rising CO_2: Light may ameliorate effects of ocean acidification on a solar-powered animal

Sue-Ann Watson

Supplementary table

S1 Table. Logistic regression Chi-squared results on the influence of CO_2 and light (PAR) on the proportion of survivors after 8 weeks. *denotes a significant result.

	df	Deviance	Residual df	Residual deviance	P value
**CO_2**	2	6.519	6	30.413	0.038*
**PAR**	2	25.659	4	4.755	0.000*
**CO_2 x PAR**	4	4.755	0	0.000	0.313
