# Supplementary material for: Giant Clams and Rising CO2: Light May Ameliorate Effects of Ocean Acidification on a Solar-Powered Animal
Source: PLoS One. 2015 Jun 17;10(6):e0128405. doi: 10.1371/journal.pone.0128405 (PMC4470504; doi:10.1371/journal.pone.0128405)
Supplement: S2 Table — *denotes a significant result. Tests were not conducted at PAR 304 as there were no deaths. (PDF) [file pone.0128405.s003.pdf]

Giant clams and rising CO_2: Light may ameliorate effects of ocean acidification on a solar-powered animal

Sue-Ann Watson

Supplementary table

S2 Table. Survival analysis results from Kaplan-Meier Log-Rank Survival Analyses on CO_2 levels at each light (PAR) condition. *denotes a significant result. Tests were not conducted at PAR 304 as there were no deaths.

Log-Rank Test	Statistic	df	P value
**Low-light PAR 35**	0.854	2	0.653
**Mid-light PAR 65**	8.935	2	0.011*
**High-light PAR 304**	n/a		
