# Supplementary material for: Giant Clams and Rising CO2: Light May Ameliorate Effects of Ocean Acidification on a Solar-Powered Animal
Source: PLoS One. 2015 Jun 17;10(6):e0128405. doi: 10.1371/journal.pone.0128405 (PMC4470504; doi:10.1371/journal.pone.0128405)
Supplement: S3 Table — *denotes a significant result. (PDF) [file pone.0128405.s004.pdf]

Giant clams and rising CO_2: Light may ameliorate effects of ocean acidification on a solar-powered animal

Sue-Ann Watson

Supplementary table

S3 Table. Pairwise multiple comparisons from Kaplan-Meier Log-Rank Survival Analysis for CO_2 levels at mid-light PAR 65. *denotes a significant result.

Comparisons	Statistic	P value
**control- vs. high-CO_2**	8.880	0.00288*
**control- vs. mid-CO_2**	4.151	0.0416*
**mid- vs. high-CO_2**	1.634	0.201
