# Supplementary material for: Giant Clams and Rising CO2: Light May Ameliorate Effects of Ocean Acidification on a Solar-Powered Animal
Source: PLoS One. 2015 Jun 17;10(6):e0128405. doi: 10.1371/journal.pone.0128405 (PMC4470504; doi:10.1371/journal.pone.0128405)
Supplement: S4 Table — LME results on total animal (soft tissues + shell) wet mass gain. (PDF) [file pone.0128405.s005.pdf]

Giant clams and rising CO_2: Light may ameliorate effects of ocean acidification on a solar-powered animal

Sue-Ann Watson

Supplementary table

S4 Table. Linear mixed effects (LME) model results. LME results on total animal (soft tissues + shell) wet mass gain.

Analysis of Variance Table

	numDF	denDF	F-value	p-value
(Intercept)	1	28	9.4596	0.0047
CO2	2	94	1.3536	0.2633
PAR	2	94	109.2171	<.0001
CO2:PAR	4	94	4.6317	0.0019

Fixed effects: pcmassgain ~ CO2 * PAR

	Value	Std.Error	DF	t-value	p-value
(Intercept)	1.5360	1.24006	28	1.238643	0.2258
CO2700	-1.9122	1.77040	94	-1.080123	0.2829
CO2950	0.5551	1.81570	94	0.305722	0.7605
PAR65	13.3043	4.28240	94	3.106738	0.0025
PAR305	157.2357	17.01488	94	9.241070	<.0001
CO2700PAR65	-11.2845	6.39301	94	-1.765127	0.0808
CO2950PAR65	-17.3029	6.92903	94	-2.497165	0.0143
CO2700PAR305	24.0054	24.37678	94	0.984766	0.3273
CO2950PAR305	-57.2323	24.06728	94	-2.378014	0.0194
