# Supplementary material for: Giant Clams and Rising CO2: Light May Ameliorate Effects of Ocean Acidification on a Solar-Powered Animal
Source: PLoS One. 2015 Jun 17;10(6):e0128405. doi: 10.1371/journal.pone.0128405 (PMC4470504; doi:10.1371/journal.pone.0128405)
Supplement: S5 Table — PCA results for shell growth morphology (shell linear dimensions: shell length, height, width and ornamentation width gains) and linear mixed effects model (LME) results on principle component (PC) 1. LME on PC2 was non-significant. (PDF) [file pone.0128405.s006.pdf]

Giant clams and rising CO_2: Light may ameliorate effects of ocean acidification on a solar-powered animal

Sue-Ann Watson

Supplementary table

S5 Table. Principle component analysis (PCA) results. PCA results for shell growth morphology (shell linear dimensions: shell length, height, width and ornamentation width gains) and linear mixed effects model (LME) results on principle component (PC) 1. LME on PC2 was non-significant.

Principle components (number of observations 131)

```
Importance of components:
                          Comp.1     Comp.2     Comp.3      Comp.4
    Standard deviation 1.9565892 0.29507569 0.22364699 0.186201642
Proportion of Variance 0.9570603 0.02176742 0.01250449 0.008667763
 Cumulative Proportion 0.9570603 0.97882774 0.99133224 1.000000000


Loadings:
             Comp.1 Comp.2 Comp.3 Comp.4
pclengthgain  0.504         0.339  0.794
pcheightgain  0.495 -0.828 -0.121 -0.235
   pcorngain  0.501  0.380  0.538 -0.561
 pcwidthgain  0.500  0.412 -0.762
```

Linear mixed effects model results for PC1

```
Analysis of Variance Table
            numDF denDF  F-value p-value
(Intercept)     1    28 6651.448  <.0001
        CO2     2    94    0.647  0.5258
        PAR     2    94  181.936  <.0001
```

```
    CO2:PAR     4    94    4.834  0.0014

Fixed effects: Comp.1 ~ CO2 * PAR
                 Value Std.Error DF   t-value p-value
 (Intercept) -1.584844 0.0328299 28 -48.27448  <.0001
      CO2700  0.062939 0.0473858 94   1.32823  0.1873
      CO2950  0.025586 0.0484929 94   0.52763  0.5990
       PAR65  0.271373 0.0936110 94   2.89894  0.0047
      PAR305  3.521392 0.3070867 94  11.46709  <.0001
 CO2700PAR65 -0.313916 0.1397754 94  -2.24586  0.0271
 CO2950PAR65 -0.362654 0.1511473 94  -2.39934  0.0184
CO2700PAR305  0.599000 0.4400010 94   1.36136  0.1767
CO2950PAR305 -0.899848 0.4345118 94  -2.07094  0.0411
```
