# Supplementary material for: Giant Clams and Rising CO2: Light May Ameliorate Effects of Ocean Acidification on a Solar-Powered Animal
Source: PLoS One. 2015 Jun 17;10(6):e0128405. doi: 10.1371/journal.pone.0128405 (PMC4470504; doi:10.1371/journal.pone.0128405)
Supplement: S6 Table — *denotes a significant result. (PDF) [file pone.0128405.s007.pdf]

Giant clams and rising CO_2: Light may ameliorate effects of ocean acidification on a solar-powered animal

Sue-Ann Watson

Supplementary table

S6 Table. Growth gain ANOVA results and pairwise multiple comparisons for experiments at each light level. *denotes a significant result.

Light	Growth gain	F or H	df	P value	Mid-CO_2	High-CO_2
**PAR 35**	Total animal mass	0.985	2,33	0.384		
	Shell length	2.025	2	0.363		
	Shell height	0.368	2,33	0.695		
	Shell ornamentation	0.423	2,33	0.659		
	Shell width	4.503	2	0.105		
**PAR 65**	Total animal mass	11.931	2	0.003*	NS	<0.05*
	Shell length	10.764	2	0.005*	<0.05*	<0.05*
	Shell height	7.218	2	0.027*	NS	<0.05*
	Shell ornamentation	4.742	2	0.093		
	Shell width	4.056	2	0.132		
**PAR 304**	Total animal mass	5.633	2,56	0.006*	NS	<0.05*
	Shell length	5.993	2,56	0.004*	NS	<0.05*
	Shell height	5.941	2,56	0.005*	NS	<0.05*
	Shell ornamentation	6.610	2,56	0.003*	NS	NS
	Shell width	3.479	2,56	0.038*	NS	NS
